# Development of global consensus sequence of HCV glycoproteins involved in viral entry

**DOI:** 10.1186/1742-4682-10-24

**Published:** 2013-04-10

**Authors:** Sobia Idrees, Usman A Ashfaq, Natasha Idrees

**Affiliations:** 1 Department of Bioinformatics and Biotechnology, Government College University (GCU), Faisalabad, Pakistan; 2 Department of Food Sciences, Government College University (GCU), Faisalabad, Pakistan

**Keywords:** HCV, Glycoproteins, Conservation, Peptides

## Abstract

**Background:**

HCV affects >170 million people worldwide and is a leading cause of liver diseases such as hepatocellular carcinoma. Each year, Pakistan reports hundreds of cases and now it has become a serious health issue. HCV has two transmembrane glycoproteins (E1 and E2) that are involved in virus entry through viral attachment, but because of their hypervariable nature they have become difficult targets for vaccine development.

**Methods:**

A total of 150 protein sequences of E1 and E2 belonging to genotypes 3a and 1a were retrieved from the NCBI protein database and were subjected to conservation and variation analysis using the multiple sequence alignment feature of the CLC workbench. A consensus sequence of each genotype of E1 and E2 was obtained and these consensus sequences were further analyzed to construct a global consensus sequence, which was used to design potentially conserved peptides.

**Results:**

From the sequence conservation analysis, highly conserved residues were identified and were used to design peptides. Only two peptides were found to be conserved in the E1 protein of genotypes 3a and 1a and a total of nine conserved peptides were designed for the HCV E2 protein of those genotypes. These designed peptides could serve as useful targets in developing new inhibitory compounds.

**Conclusion:**

This study was designed to perform conservation and variability analysis of HCV glycoproteins and to find potentially conserved peptides among genotypes 3a and 1a (the most prevalent genotypes in Pakistan) that could serve as useful targets in the development of novel inhibitory compounds, thus reducing the threat of HCV infection in Pakistan.

## Background

Chronic infection with hepatitis C virus (HCV) affects >170 million individuals, approximately 3% of the world population, and is responsible for approximately 350,000 deaths every year [1]. HCV has become a leading cause of liver diseases such as hepatocellular carcinoma. It is a small, single-stranded, positive strand RNA virus encoding structural and non-structural proteins. It has many genetic variants, an important factor to consider in drug development. There are six known major genotypes and >100 subtypes of HCV [1,2] and owing to this strain variability, no vaccine has been developed to date; the currently approved treatment for HCV is pegylated interferon α (PEG-INF α) in combination with ribavirin and Boceprevir/Telaprevir.

Pakistan has become a significant reservoir of HCV and each year hundreds of people become infected. Prevalence analysis has revealed that the most prevalent HCV genotype in Pakistan is 3a, except in Balochistan where the most frequent subtype is 1a [3]. Among the HCV structural proteins, the HCV glycoproteins (E1 and E2) are both hypervariable transmembrane are present on the surface of the virus and are extremely involved in virus attachment with host cell through cell receptors [4,5]. Envelope protein E1 has a C-terminal domain involved in membrane permeability changes and membrane association [3]. Envelope protein E2 contains up to 11 N-linked glycosylation sites and is involved in viral entry by interacting with an extracellular loop of human CD81, scavenger receptor class B type 1 (SRB-1), high density lipoprotein (HDL) binding molecule and mannose binding proteins (DC-SIGN and L-SIGN) [6-12]. Both glycoproteins are important in viral entry but because of their hypervariable nature it is difficult to design vaccines or inhibitory compounds against them. Therefore, this study was conducted to perform a sequence analysis of E1 and E2 among genotypes 3a and 1a to determine conserved peptides in HCV that could be useful targets in the design of novel inhibitory compounds, thus reducing threats of HCV in Pakistan.

## Results

### Global consensus sequence development

**Figure 1:**
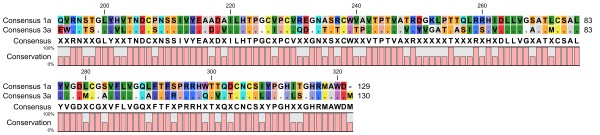
**Multiple sequence alignment showing global consensus sequence of HCV E1 protein of genotypes 3a and 1a isolated from different regions of the world.** The global consensus sequence is shown at the bottom of the alignment; identical residues are represented by (.) and ambiguous symbols are represented by (X).

**Figure 2:**
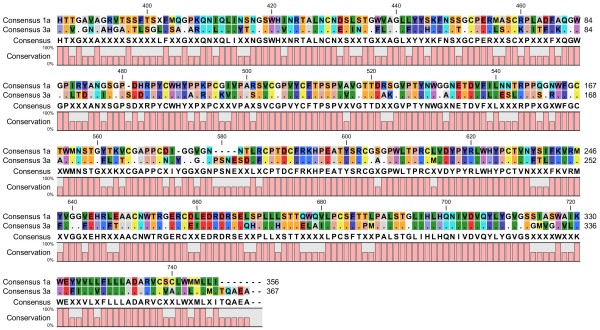
**Multiple sequence alignment showing global consensus sequence of HCV E2 protein of genotypes 3a and 1a isolated from different regions of the world.** The global consensus sequence is shown at the bottom of the alignment; identical residues are represented by (.) and ambiguous symbols are represented by (X).

To design effective peptides, a consensus-based approach was used. A global consensus sequence was constructed using the multiple alignment feature of the CLC Workbench for HCV E1 (Figure 1) and E2 (Figure 2) proteins. The global consensus sequence is shown at the base of the alignments in Figures 1 and 2.

### Peptide design

Sequences of HCV E1 and E2 of genotypes 3a and 1a were retrieved from the NCBI protein database and were subjected to conservation analysis using the multiple sequence alignment feature of the CLC workbench. From the highly conserved residues of E1, short peptides of 8–25 amino acids were designed (Table 1), and from the highly conserved regions of E2, similarly short peptides were designed using the same criteria as for E1 (Table 2). These peptides are conserved among the 1a and 3a genotypes, so they could be useful for designing peptide-based vaccines and inhibitory compounds.

**Table 1:** Predicted peptides for HCV E1 conserved in 1a and 3a genotypes

**Sr. No.**	**Position**	**Peptide**	**Length**	**Molecular weight (g/mol)**	**Extinction coefficient (cm**^**-1 **^**M**^**-1**^**)**	**Composition of (Leu, Val, Ile, Met, Phe, and Trp)**
1	209-216	NSSIVYEA	8	882	1280	25%
2	272-279	CSALYVGD	8	827	1400	25%

**Table 2:** Predicted peptides for HCV E2 conserved in 1a and 3a genotypes

**Sr. No.**	**Position**	**Peptide**	**Length**	**Molecular weight (g/mol)**	**Extinction coefficient (cm**^**-1 **^**M**^**-1**^**)**	**Composition of (Leu, Val, Ile, Met, Phe, and Trp)**
1	423-430	NRTALNCN	8	905	120	12.5%
2	502-515	SVCGPVYCFTPSPV	14	5.24	1615	22%
3	524-531	GVPTYNWG	8	893	6970	25%
4	587-604	CPTDCFRKHPEATYSRCG	18	2071	1640	6%
5	606-613	GPWLTPRC	8	929	5810	25%
6	615-629	VDYPYRLWHYPCTVN	15	1926	9650	27%
7	648-658	AACNWTRGERC	11	1266	5930	10%
8	689-713	PALSTGLIHLHQNIVDVQYLYGVGS	25	2695	2560	36%
9	729-738	FLLLADARVC	10	1120	120	50%

### Phylogenetic analysis

**Figure 3:**
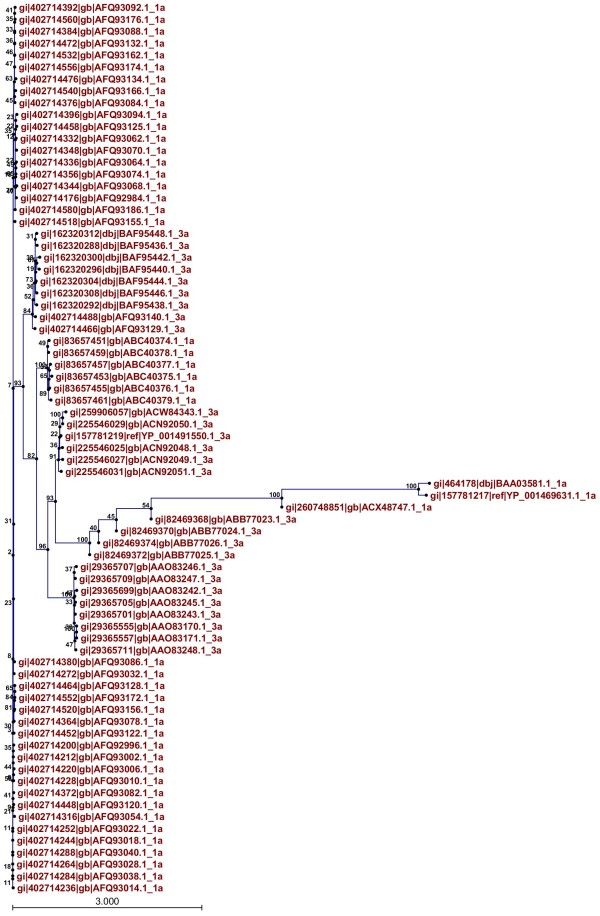
Phylogenetic tree showing evolutionary relationships among HCV E1 proteins of genotype 3a with genotype 1a isolated from different regions of the world.

**Figure 4:**
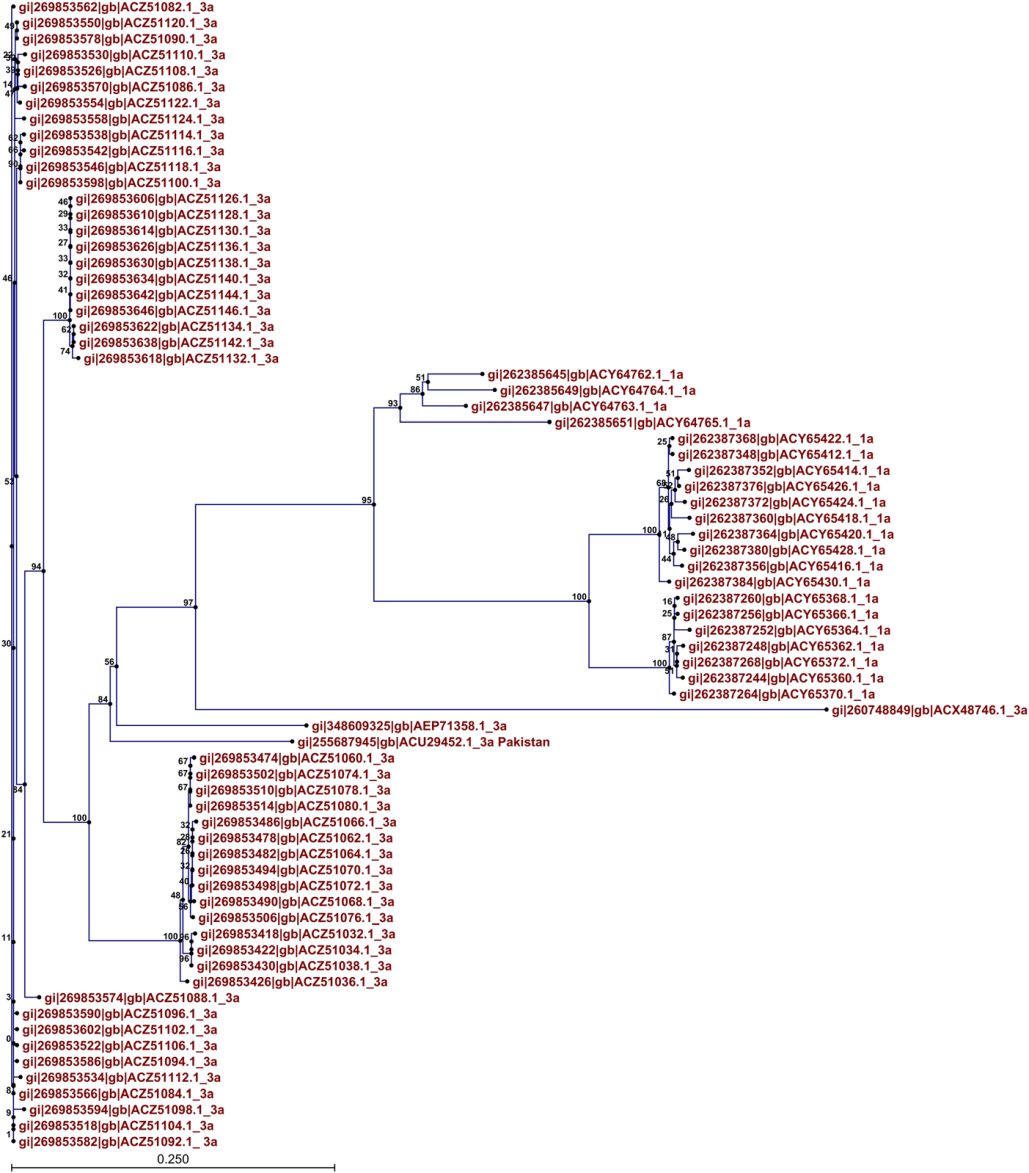
Phylogenetic tree showing evolutionary relationships among HCV E2 proteins of genotype 3a with genotype 1a isolated from different regions of the world.

Phylogenetic trees of both proteins showed clusters built on the basis of evolutionary relatedness. It can be inferred that envelope proteins of genotype 3a isolated from different regions of the world are evolutionarily related and are also related to 1a from different countries. It can also be inferred that genotypes 1a and 3a share a common ancestry. Therefore it can be concluded that HCV E1 and E2 are evolutionarily related to 3a in other countries and are also related to genotype 1a. The evolutionary tree of E1 protein is shown in Figure 3 and that of HCV E2 protein is shown in Figure 4.

## Discussion

HCV infection has become a serious health problem worldwide but owing to its six genotypes and more than 100 subtypes it is elusive in vaccine development. HCV has a 9.6kB genome that encodes 10 proteins in the order NH_2_-Core-E1-E2-p7-NS2-NS3-NS4A-NS4B-NS5A-NS5B-COOH [2]. Among these proteins, E1 and E2 are involved in viral entry as they are required for virus attachment to receptors on the host cell. Therefore these two proteins could serve as useful targets in in the design of novel inhibitory compounds. However, because of their hypervariable nature, targeting these proteins is difficult.

Keeping these points in mind, this study was designed to perform conservation and variation analysis on both E1 and E2 sequences isolated from different regions of the world and, among all the reported HCV genotypes, sequences related to only genotypes 3a and 1a were retrieved and studied. These genotypes (3a and 1a) were selected as they are reported to be the most prevalent in Pakistan. Analyzing these sequences could help in development of inhibitory compounds with the potential not only to reduce viral titers but also contribute to reducing the threat of HCV in the Pakistani population. *In-silico* analysis of both glycoproteins revealed conserved and variable regions. From the highly conserved regions of both proteins, conserved peptides were designed that could potentially serve as targets for vaccine or inhibitory compound development.

Moreover, phylogenetic analysis was conducted to find the evolutionary relationship between the HCV E1 and E2 genotype 3a of Pakistani origin and E1 and E2 sequences of genotypes 3a and 1a. In the case of E1 protein, genotype 1a appeared at the root of the tree and it can be inferred that genotype 3a has evolved from it. In the case of E2 protein, genotype 3a appeared at the root of the tree and it can be inferred that genotype 1a has evolved from it. The HCV envelope proteins (E1/E2) in Pakistan are highly evolutionarily related to 1a and 3a from other countries.

## Conclusion

Genotype 3a is reported as the most prevalent genotype in Pakistan except in Balochistan where the most prevalent genotype is 1a. This study was designed to identify potentially conserved peptides in the two hypervariable proteins, E1 and E2, of HCV. From the conservation analysis, highly conserved and variable regions were revealed and from the highly conserved regions peptides were designed that could serve as useful targets for developing novel inhibitory compounds against both 3a and 1a genotypes, potentially protecting the Pakistani population against the threat of HCV infection.

## Methods

### Global consensus sequence development

A total of 150 HCV E1 and E2 sequences belonging to genotypes 3a and 1a were retrieved from the NCBI protein database. Consensus sequences of E1 and E2 proteins for each genotype were constructed using the multiple sequence analysis feature of the CLC workbench. These consensus sequences were further used to construct global consensus sequences for E1 and E2.

### Peptide design

The consensus sequences of E1 belonging to genotypes 3a and 1a were aligned to identify the global consensus sequence of E1 in Pakistan and other countries. A global consensus sequence for E2 protein was found using the same strategy. These global consensus sequences could help in designing effective conserved peptides against genotypes 3a and 1a. Short peptide sequences were derived from the highly conserved regions of HCV E1 and E2. As the HCV glycoproteins are highly variable and are required for viral entry, it is important to identify conserved peptides that could serve as the best targets for potential inhibitors and vaccines. In designing peptides for successful antibody production, many factors were considered. These included amino acid composition and peptide length, which can influence correct assembly, purification, and subsequent solubilization. (1) The peptide length criterion was set at 8–25. Typically, a 10–15 residue length is used for raising antisera. (2) The hydrophobic amino acid content (Leu, Val, Ile, Met, Phe, and Trp) was not allowed to exceed 50% in the designed peptide. Conserved peptides were designed from the global consensus sequence while considering all these factors.

### Phylogenetic analysis

HCV E1 and E2 sequences from all over the world were analyzed to establish their evolutionary relationships using the CLC workbench. The phylogenetic analysis feature of the workbench was used to construct phylogenetic trees for E1 and E2 proteins using the neighbor-joining method with a bootstrap value of 100.

### Future directions

In future, we aim to analyze these peptides further to indicate methods for blocking HCV entry.

## Competing interests

The authors declare that they have no competing interests.

## Authors’ contributions

UAA designed the study, SI performed the sequence analysis, NI helped in manuscript writing. All authors read and approved the final manuscript.
